# Growth of *Pleurotus Ostreatus* on Different Textile Materials for Vertical Farming

**DOI:** 10.3390/ma12142270

**Published:** 2019-07-15

**Authors:** Julia Helberg, Michaela Klöcker, Lilia Sabantina, Jan Lukas Storck, Robin Böttjer, Bennet Brockhagen, Franziska Kinzel, Anke Rattenholl, Andrea Ehrmann

**Affiliations:** Faculty of Engineering and Mathematics, Bielefeld University of Applied Sciences, 33619 Bielefeld, Germany

**Keywords:** textile materials, knitted fabrics, agar, *Pleurotus ostreatus*, vertical farming

## Abstract

The mycelium of the edible mushroom *Pleurotus ostreatus* can be used for diverse technical applications, such as packaging materials or wastewater treatment, besides the more obvious use for nutrition. While *P. ostreatus* usually grows on sawdust, wood or similar materials, a former study investigated mycelium growth on different nanofiber mats. Here, we report on growing *P. ostreatus* on fabrics knitted from different materials, enabling the use of this mushroom in textile-based vertical farming. Our results underline that *P. ostreatus* grows similar on natural fibers and on synthetic fibers. The agar medium used to provide nutrients was found to support mycelium growth optimally when applied by dip-coating, suggesting that, in this way, *P. ostreatus* can also be grown on vertically aligned textile fabrics for vertical farming.

## 1. Introduction

Textile materials can be used for a broad variety of applications, from clothing to home textiles to medical or technical textiles. Another large area in which textile fabrics are used is agriculture. Agro-textiles are often used to cover growing plants, for example, to support outdoor lettuce growth [1,2], early potato plants [3] or spinach [4]. These agro-textiles are often woven but can also be warp or weft knitted [5]. Typical materials are polyethylene [6,7], polyester, polyamide [5] and other chemical fibers, partly finished with chemicals to protect plants from vectors and other contaminations [7,8,9,10].

The idea of letting plants grow on textile fabrics instead of underneath them, however, is relatively new [11], while the more general idea of vertical farming on diverse substrates or in hydroponics is already applied. Vertical farming means growing plants, especially edible ones, in so-called farm-scrapers with a closed water recycling system. It can also be regarded as growing plants on walls, that is, on really vertical substrates, to reduce the necessary area for the desired amount of food. Vertical farming recently belongs to the topics of high interest in research and development due to the increasing problem of hunger and malnutrition with increasing population growth especially in the poorest countries [12,13] but also due to the increasing risks of outdoor farming in view of climate change and more and more extreme weather conditions [14].

Interestingly, mushroom growth in vertical farming cannot be found yet in the scientific literature although edible mushrooms are usually protein-rich [15], are regarded as antioxidative and antimicrobial [16], making them interesting as food [17] as well as in diverse technical applications [18]. Only a few studies have investigated the influence of textile [19,20] and other substrates on the mycelium or fruit body growth [21,22], while a significant effect of the substrate on mycelium growth has been shown [20,23].

Here, we report on the first study in which the edible mushroom *Pleurotus ostratus* [24] is grown on knitted fabrics prepared from different materials, using different loop dimensions to investigate the influence of substrate material and structure on mycelium growth. In addition, different possibilities for adding nutrient agar were investigated to enable a future application in vertical farming, allowing edible mushrooms to grow on the smallest spaces in cities or inside buildings. Finally, the possibility to use such bio-based composites for technical purposes is discussed.

## 2. Materials and Methods

The knitted fabrics were produced on a hand flat knitting machine Silver Reed SK 280 (Knittax, Darmstadt, Germany) with gauge E5.6 (needle distance 4.5 mm), using the machine-specific stitch dimensions 2, 4, 6, 8 and 10 to produce single-jersey fabrics. The following yarn materials (all purchased from Andreas Hoffmann Garnhandel & Textilspulerei, Engstingen, Germany) were used: polyacrylonitrile grey/white (yarn count: Nm 22/2, meaning 2 threads of a yarn with a linear weight of 1 g per 22 m); cotton 75%/linen 25% light grey (Nm 10/3); pure cotton dark grey (Nm 3/1); polyacrylonitrile (acrylic) 70%/wool 30% dusty pink (Nm 28/2); and acrylic 50%/polyester 50% (Nm 30/2). These yarns were chosen to investigate the difference between pure synthetic fibers (polyacrylonitrile, polyester), pure natural fibers (cotton, linen) and a mixture of both (acrylic/wool).

The knitted fabrics were washed at 90 °C in a household washing machine Maxx 6 Exxpress (Robert Bosch GmbH, Gerlingen, Germany), using the color detergent Ariel Colorwaschmittel Pulver (Procter & Gamble Service GmbH, Schwalbach am Taunus, Germany) and a second time washed without detergent at the same temperature to clean the fabrics from possible residues from the spinning process. Afterwards, the samples were dried lying on a flat area at room temperature.

For sterilization of all materials, they were autoclaved for 20 min at 121 °C in an autoclave Systec-VX75 (Systec, Linden, Germany).

Malt extract agar was used as nutrient medium for mycelium growth and was produced from 1 L deionized water, 24 g agar (agar-agar Kobe I, Roth, Karlsruhe, Germany), 20 g barley malt extract (Lindenmeyer GmbH & Co. KG, Weinsberg, Germany) and 1 g peptone (peptone water 77185, Sigma-Aldrich GmbH, Steinheim, Germany). Afterwards, the fluid solution was autoclaved and poured into 8.5 cm diameter petri dishes (VWR, Darmstadt, Germany). The knitted fabrics were also partly dip-coated in warm agar. All petri dishes—containing pure knitted fabrics on an agar plate, dip-coated knitted fabrics on an agar plate and dip-coated knitted fabrics without additional agar plate—were inoculated with the sterile liquid mycelium “oyster mushroom culture XXL, BIO” (purchased from Mushrooms & Equipment Shop, Münster, Germany), using 1 drop of liquid mycelium culture per petri dish, and afterwards sealed with Parafilm (Pechiney Plastic Packaging, Chicago, IL, USA). Mycelium growth was observed at the optimal growth temperature of 28 °C [25] in a relative humidity of 30–35 % without artificial light (i.e., nearly in complete darkness) in a universal heating cabinet UN 75 (Memmert, Schwabach, Germany). Each experiment was performed with n = 5.

To measure the number of stitches per area, course and wale density as well as for optical investigation of mycelium growth, a digital microscope VHX-600K (Keyence, Neu-Isenburg, Germany) was used. More detailed images were taken with a scanning electron microscope (SEM) Zeiss 1450VPSE (Oberkochen, Germany). For the examination of the mycelium growth area, photographic images were taken from a constant distance with a Galaxy S9 (Samsung, Seoul, South Korea) and analyzed with the software ImageJ 1.51j8 (from National Institutes of Health, Bethesda, MD, USA) by counting the pixels in the mycelium area and calculating an area and a corresponding diameter from this number.

Knitted fabric thicknesses were measured by a digital thickness gauge J-40-T (Wolf-Messtechnik GmbH, Freiberg, Germany), areal weights by an analytical balance SE-202 (VWR International GmbH, Darmstadt, Germany).

## 3. Results and Discussion

During knitting, some combinations of yarns and stitch dimensions were found not to be producible, for example, the smallest stitches (“2” and “4”) with the cotton yarn. The acrylic/wool and acrylic/polyester yarns, on the other hand, were relatively fine and thus complicated to use.

**Figure 1:**
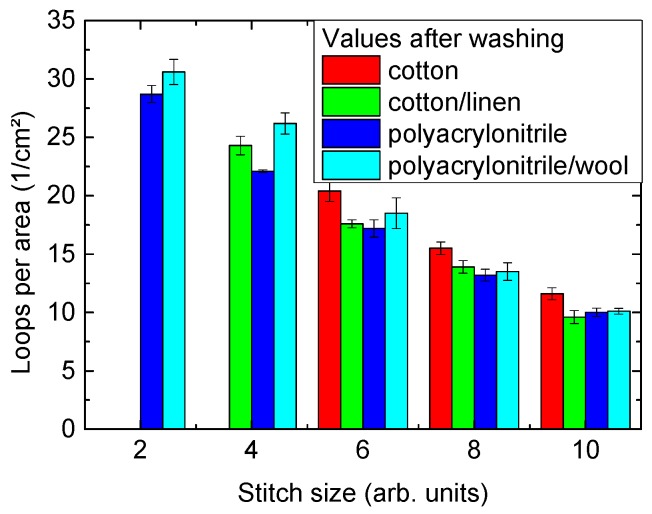
Numbers of loops per area, measured after washing using microscopic images of areas (11 mm × 15 mm). The stitch sizes are given by the machine definition, ranging from 1–10. The resulting structural parameters of the knitted fabrics can be found below.

Another problem was found during washing—here, both yarns containing cotton showed certain shrinkage, while the polyacrylonitrile/polyester and to a certain extent also the polyacrylonitrile/wool fabrics lost their macroscopic shape, that is, they became much longer and slimmer. The acrylic/polyester fabrics showed dimensional changes up to a factor of nearly 2, with strongly varying deformations so that the fabrics prepared from this yarn were not taken into account for the tests. The loops per area of the other fabrics, measured after washing, are depicted in Figure 1. As expected, the smaller stitch dimensions, defined by the machine settings, correspond to higher numbers of loops per area, with a factor of nearly 3 between smallest and largest numbers of loops per area for both yarns containing polyacrylonitrile and approx. a factor of 2 for the other two yarns, indicating the large range of stitch dimensions and thus of open areas between the yarn paths, possibly influencing mycelium growth.

Next, Table 1, Table 2, Table 3, Table 4, Table 5 and Table 6 show more parameters defining the knitted fabrics’ parameters, allowing for characterization of the fabrics’ structures. As indicated here, a broad range of parameters was tested in this study, allowing for estimating their influence on mycelium growth.

**Figure 2:**
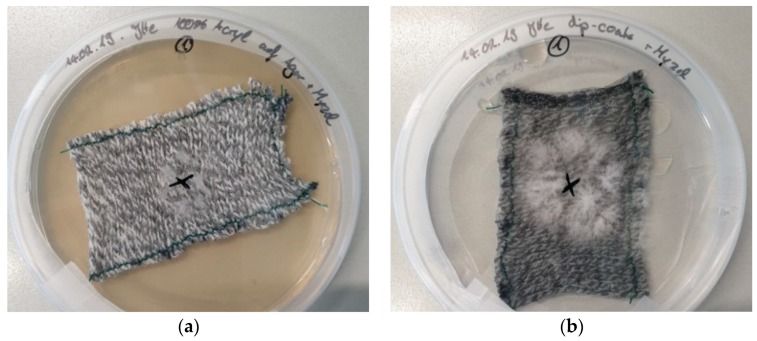

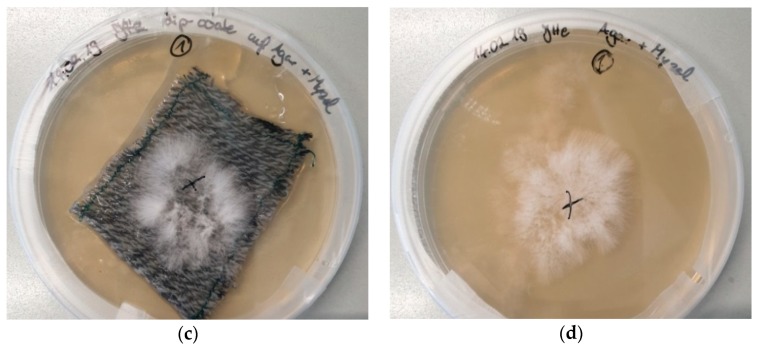
*Pleurotus ostreatus* mycelium grown on different substrates, 4 days after inoculation: (**a**) acrylic knitted fabric on agar plate; (**b**) acrylic knitted fabric dip-coated with agar; (**c**) acrylic knitted fabric dip-coated with agar on agar plate; (**d**) agar plate.

In the first experiments, how the knitted fabric should ideally be combined with the agar serving as nutrient medium was tested. Figure 2 depicts exemplary results for the pure acrylic fabric in different combinations with agar, compared with a pure agar plate as a reference. All images were taken 4 days after inoculation.

It is clearly visible that mycelium growth is slowest on the pure fabric placed on an agar plate (Figure 2a) which can be attributed to the reduced contact between the liquid mycelium culture and the agar. The other three cases show a significantly stronger growth, mostly on an approximately circular area as seen on nanofiber mats [20] but also with extensions from this expected area, most probably due to undesired flowing of the initial drop of liquid mycelium culture from the position marked by an “x.”

**Figure 3:**
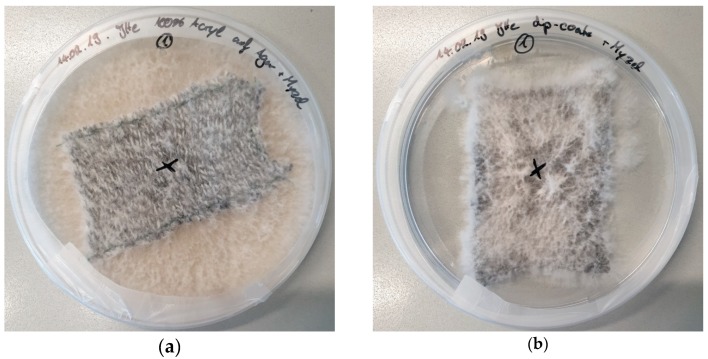

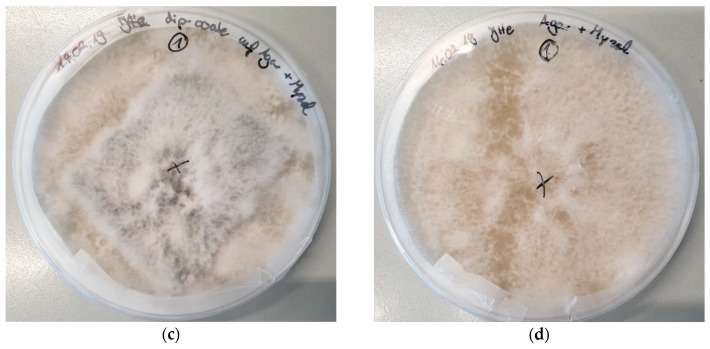
*Pleurotus ostreatus* mycelium grown on different substrates, 12 days after inoculation: (**a**) acrylic knitted fabric on agar plate; (**b**) acrylic knitted fabric dip-coated with agar; (**c**) acrylic knitted fabric dip-coated with agar on agar plate; (**d**) agar plate.

In all cases, the substrates were completely covered with mycelium after 11–12 days. Figure 3 depicts photographic images of the samples shown in Figure 2 after 12 days.

It is clearly visible that the mycelium grows thickest on pure agar (Figure 3d) as well as on the combination with a dip-coated fabric on an agar plate (Figure 3c), while the pure fabric on the agar plate results in only weak growth. This shows that dip-coating the fabric with agar is essential to promote mycelium growth, the opposite of former tests with nanofiber mats [20] in which it was sufficient to place the textile substrates on agar plates. Here, however, the relatively thick knitted fabrics prevent the mycelium from reaching the agar plate below and thus disturb the growth process as long as they are not soaked with agar themselves.

**Figure 4:**
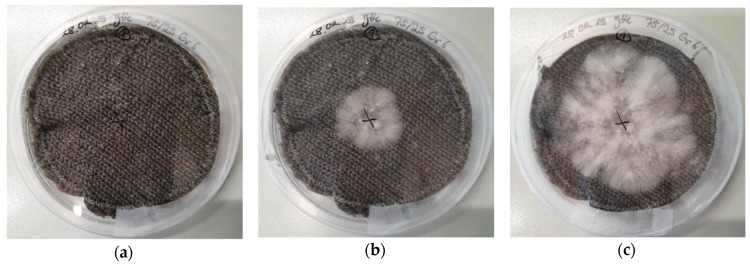

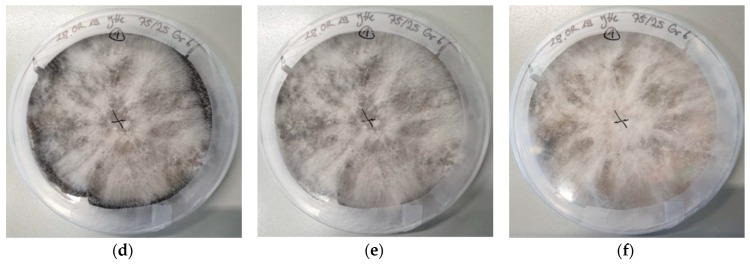
*Pleurotus ostreatus* mycelium grown on a cotton/linen fabric with stitch size 6 after different growth times: (**a**) 0 days; (**b**) 4 days; (**c**) 6 days; (**d**) 7 days; (**e**) 8 days; (**f**) 15 days.

As the main aim of this study was to investigate whether textile substrates may be used in the vertical farming of mushrooms, the subsequent examinations concentrated on the dip-coated textile fabrics without an additional agar plate beneath, which would be complicated to implement in a vertical setup. To enable comparison of the growth processes on the different knitted fabrics, they were cut into the round shapes of the petri dishes or slightly smaller due to shrinkage during washing; the maximum diameter of the mycelium is thus given by the 8.5 cm diameter of the petri dishes (for cotton and cotton/linen) or by 7 cm (for acrylic and acrylic/wool). Exemplarily, Figure 4 depicts the growth process on a knitted fabric from cotton/linen, produced with average stitch size 6.

Starting around the inoculation position, marked with an “x,” the mycelium grows quite radially, until after 8 days (Figure 4e) the whole knitted fabric is covered with mycelium. Afterwards, the mycelium thickness further increases (Figure 4f), as can be seen by the brighter mycelium in Figure 4f, as compared to the thinner mycelium in Figure 4e through which the dark knitted fabric is more visible.

**Figure 5:**
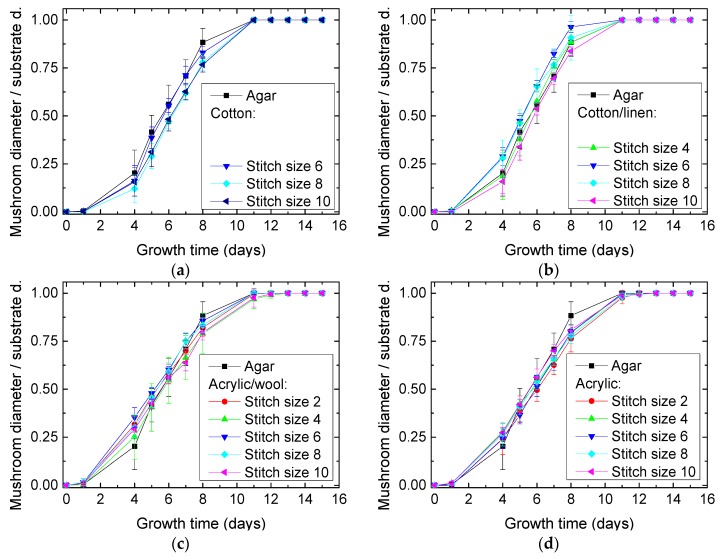
Growth process (diameters normalized to the substrate diameters) of *Pleurotus ostreatus* mycelium on dip-coated substrates from different materials: (**a**) cotton; (**b**) cotton/linen; (**c**) acrylic/wool; (**d**) acrylic.

To examine the influence of the stitch dimensions, Figure 5 shows the growth processes on the different textile substrates under examination. During the first few days, that is, in the so-called lag phase, the mycelium adapts to the new substrates on which it was inoculated and does not grow [26]. In the subsequent exponential (or log) phase, the fastest growth occurs. Finally, this phase is stopped, often by the depletion of nutrients in the medium, here by the borders of the knitted fabrics.

The growth in the exponential phase is of great interest in terms of comparing the different stitch sizes and fabrics. Unexpectedly, in all cases no significant influence of the stitch sizes nor thicknesses or the textile substrate were visible, opposite to the results of a former study growing *P. ostreatus* mycelium on nanofiber mats [20].

**Figure 6:**
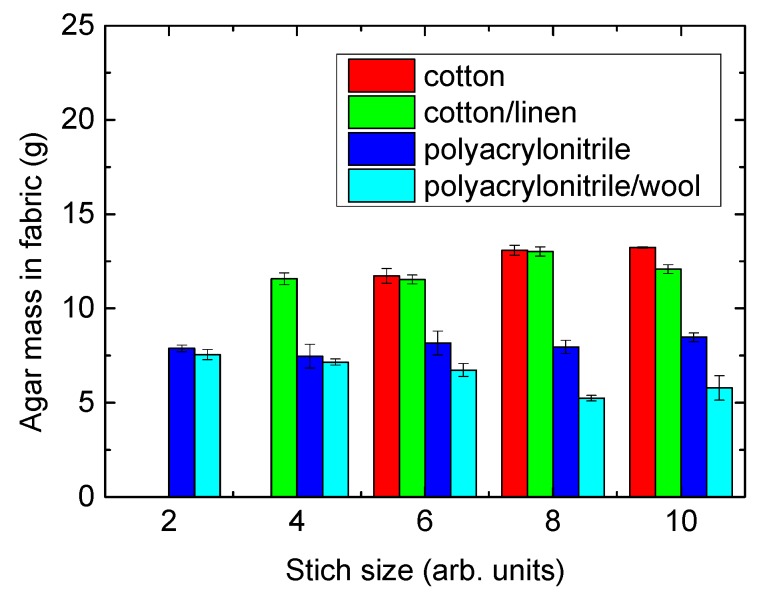
Amount of agar absorbed in the different fabrics.

This can be explained by enough malt agar being available during the growth process under examination so that the substrate does not have to be used as another possible source of nutrients. The amount of medium, which is linearly correlated with the available nutrients, is depicted in Figure 6.

Here it is clearly visible that cotton and cotton/linen always absorb significantly more agar than acrylic or acrylic/wool. On the other hand, the amount of agar in the acrylic/wool fabrics decreases with the stitch size. This finding suggests future experiments with other knitted structures, such as plush, to create highly absorbing acrylic-based knitted fabrics which enable long growth processes without the necessity of using the textile structure as an additional nutrient.

**Figure 7:**
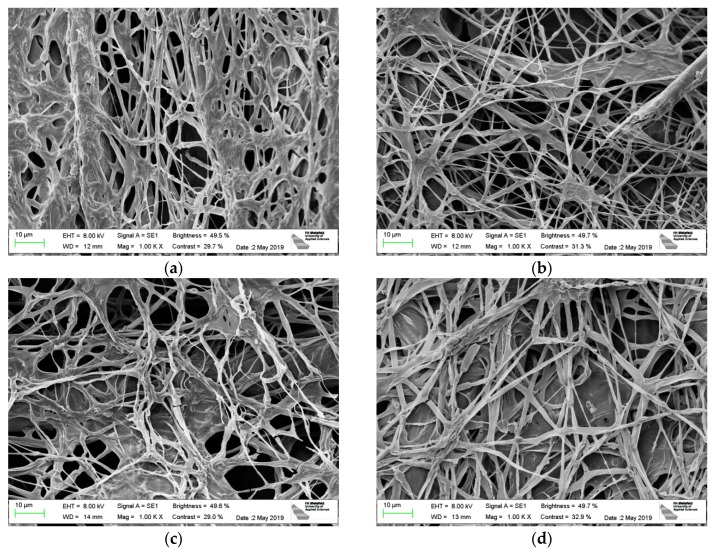
Scanning electron microscope (SEM) images of *Pleurotus ostreatus* mycelium on dip-coated substrates from different materials, knitted with stitch size 6: (**a**) cotton; (**b**) cotton/linen; (**c**) acrylic/wool; (**d**) acrylic.

The morphology of the mycelium fibers, grown on different knitted fabrics, is depicted in Figure 7. While in a former investigation on nanofiber mats, severely different morphologies were found, depending on the substrate [20]; here the morphologies look similar on all knitted fabrics. On each substrate, denser and less densely covered areas are visible as well as thinner and thicker fibers, partly grown together with neighboring fibers. In none of the samples, a macroscopic orientation of the mycelium fibers can be recognized, as it was the case on nanofiber mats [20].

While the main aim of this study was the utilization of mushrooms in vertical farming on textile substrates, the technical possibility of using this technique for the production of composites shall also be discussed in brief.

**Figure 8:**
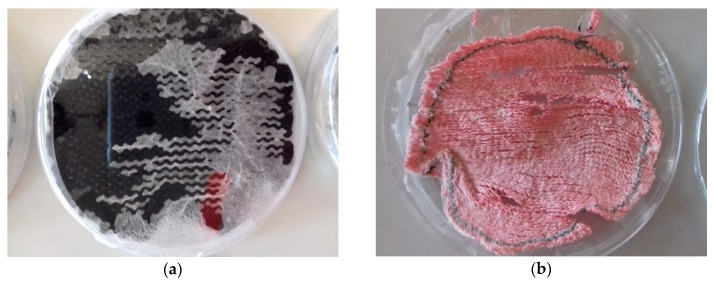
Mycelium after finished experiments: (**a**) rear side of cotton fabric before drying; (**b**) dried acrylic/wool sample.

As Figure 8a reveals, the mycelium tended to grow through the knitted fabrics but usually did not complete this process during the 15 days of growth examined here. For longer growth times, however, it can be expected to completely fill the areas between the yarns of the knitted fabrics, in this way forming a composite with the knitted fabric. Such mycelium-based composites are not only interesting due to their possible use in circular economy, if natural fibers are used but also since their mechanical properties can be tailored by varying substrate, fungal species and processing techniques [27]. Warp or weft knitted spacer fabrics can be expected to be of special interest since they can be inoculated between front and rear side, so that the mycelium can grow inside and fill it completely.

To investigate the possible applicability of *P. ostreatus* in forming composites with knitted fabrics, a preliminary test was performed to dry the mycelium at 60 °C for 48 h, in this way devitalizing it. As visible in Figure 8b, the resulting composite is already quite stiff, suggesting a potential usability in technical applications. It should be mentioned that the visible breaks occurred while taking the composite out of the petri dish to which the dried agar solution adhered strongly and cannot be attributed to the drying process itself. Investigations of the mechanical properties of such composites will thus be performed in a future test series, after trying to separate composites from agar without agar residues.

## 4. Conclusions

Mycelium of *Pleurotus ostreatus* was grown on different textile fabrics in the presence of nutrient agar. No influence of the textile material or the stitch size on the growth rate or the mycelium morphology was visible, contrary to a former investigation of mycelium growth on nanofiber mats [20], indicating that the nutrient agar absorbed in the textile fabrics was sufficient for the growth duration under examination.

After drying the composites at 60 °C and, in this way, devitalizing the mycelium, a rigid composite was generated, suggesting further experiments with defined drying and pressing processes to create bio-based sustainable composites with the desired mechanical properties.

## Figures and Tables

**Table 1:** Thicknesses of knitted fabrics under examination in this study.

Stitch Size	Cotton	Cotton/Linen	Polyacrylonitrile	PAN/Wool
2	-	-	1.77 mm	1.19 mm
4	-	2.02 mm	1.80 mm	1.16 mm
6	2.75 mm	2.04 mm	1.84 mm	1.13 mm
8	2.90 mm	2.06 mm	1.86 mm	1.12 mm
10	2.98 mm	2.08 mm	1.90 mm	1.05 mm

**Table 2:** Areal weights of knitted fabrics under examination in this study.

Stitch Size	Cotton	Cotton/Linen	Polyacrylonitrile	PAN/Wool
2	-	-	222 g/m^2^	120 g/m^2^
4	-	553 g/m^2^	200 g/m^2^	114 g/m^2^
6	701 g/m^2^	496 g/m^2^	192 g/m^2^	107 g/m^2^
8	660 g/m^2^	430 g/m^2^	170 g/m^2^	93 g/m^2^
10	560 g/m^2^	378 g/m^2^	148 g/m^2^	79 g/m^2^

**Table 3:** Apparent densities of knitted fabrics under examination in this study.

Stitch Size	Cotton	Cotton/Linen	Polyacrylonitrile	PAN/Wool
2	-	-	125 kg/m^3^	101 kg/m^3^
4	-	274 kg/m^3^	111 kg/m^3^	98 kg/m^3^
6	255 kg/m^3^	243 kg/m^3^	104 kg/m^3^	95 kg/m^3^
Stitch size 8	228 kg/m^3^	209 kg/m^3^	91 kg/m^3^	83 kg/m^3^
10	188 kg/m^3^	182 kg/m^3^	78 kg/m^3^	75 kg/m^3^

**Table 4:** Stitch lengths of knitted fabrics under examination in this study.

Stitch Size	Cotton	Cotton/Linen	Polyacrylonitrile	PAN/Wool
2	-	-	9.4 mm	9.9 mm
4	-	11.3 mm	11.2 mm	11.3 mm
6	12.7 mm	12.5 mm	12.7 mm	12.5 mm
8	14.2 mm	14.8 mm	14.8 mm	14.2 mm
10	17.5 mm	16.6 mm	17.0 mm	15.7 mm

**Table 5:** Course density of knitted fabrics under examination in this study.

Stitch Size	Cotton	Cotton/Linen	Polyacrylonitrile	PAN/Wool
2	-	-	6.1/cm	6.0/cm
4	-	6.4/cm	4.9/cm	5.7/cm
6	5.1/cm	5.5/cm	4.0/cm	5.0/cm
8	4.3/cm	4.8/cm	3.3/cm	4.1/cm
10	4.0/cm	4.0/cm	2.7/cm	3.6/cm

**Table 6:** Wale density of knitted fabrics under examination in this study.

Stitch Size	Cotton	Cotton/Linen	Polyacrylonitrile	PAN/Wool
2	-	-	4.7/cm	5.1/cm
4	-	3.8/cm	4.5/cm	4.6/cm
6	4.0/cm	3.2/cm	4.3/cm	3.7/cm
8	3.6/cm	2.9/cm	4.0/cm	3.3/cm
10	2.9/cm	2.4/cm	3.7/cm	2.8/cm
